# Low-Input Estimation of Site-Specific Lime Demand Based on Apparent Soil Electrical Conductivity and In Situ Determined Topsoil pH

**DOI:** 10.3390/s19235280

**Published:** 2019-11-30

**Authors:** Moritz von Cossel, Harm Druecker, Eberhard Hartung

**Affiliations:** 1 Department of Biobased Products and Energy Crops (340b), Institute of Crop Science, University of Hohenheim, Fruwirthstr. 23, 70599 Stuttgart, Germany; 2 Institute of Agricultural Engineering, Kiel University, Olshausenstr. 40, 24098 Kiel, Germany; harm.druecker@lwk-niedersachsen.de

**Keywords:** liming, precision farming, EM38, soil acidity, digital soil mapping

## Abstract

Site-specific liming helps increase efficiency in agricultural production. For adequate determination of the lime demand, a combination of apparent soil electrical conductivity (EC_a_) and topsoil pH can be used. Here, it was hypothesized that this can also be done at low-input level. Field measurements using the EM38 MK I (Geonics, Canada) were conducted on three experimental sites in north Germany in 2011. The topsoil pH was measured based on two approaches: on the field using a handheld pH meter (Spectrum-Technologies Ltd., Bridgend, UK) with a flat electrode (in situ), and in the lab using standard equipment (ex situ). Both soil EC_a_ (0.4–35.9 mS m^−1^) and pH (5.13–7.41) were heterogeneously distributed across the sites. The same was true of the lime demand (−1.35–4.18 Mg ha^−1^). There was a significant correlation between in situ and ex situ determined topsoil pH (r = 0.89; *p* < 0.0001). This correlation was further improved through non-linear regression (r = 0.92; *p* < 0.0001). Thus, in situ topsoil pH was found suitable for map-overlay with EC_a_ to determine the site-specific lime demand. Consequently, the hypothesis could be confirmed: The combined use of data from EM38 and handheld pH meters is a promising low-input approach that may help implement site-specific liming in developing countries.

## 1. Introduction

**Figure A1:**
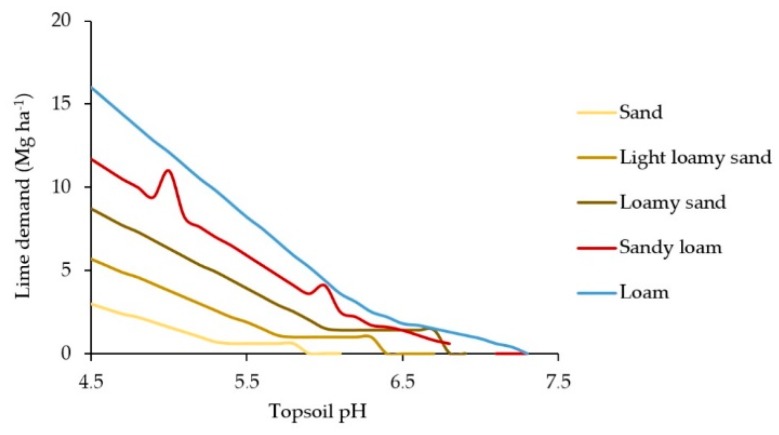
Soil-specific lime demand according to the topsoil pH (adapted from [46]).

Liming, the application of finely ground limestone or CaCO_3_, is an indispensable practice for maintaining productivity on arable lands in humid climate zones due to soil acidification following permanent leaching of carbonate fractions [1,2]. It helps to ensure long-term soil fertility and thus, improves the growth conditions for arable crops, including pastures and grassland regimes with a lower pH threshold than for arable crops. On arable sites, liming should be applied once every five to six years or whenever it is required due to pH-levels falling below the site-specific pH thresholds, an increased risk of clubroot (*Plasmodiophora brassicae* Woronin, 1877) onset, or for other reasons [3]. For effective application on the topsoil pH, several tons of lime are required per hectare (Figure A1). The cost of lime ranges between 30 and 36 € Mg^−1^ depending on the type of lime fertilizer [4]. Additionally, further costs arise from both the transport of the lime to the field and its application on the field [5]. Since the amount of lime used in agriculture has increased from 2.67 to 2.94 Tg from 2017 to 2018 in Germany [6] lime fertilization is an economically significant part of agriculture. Therefore it is critical that liming becomes less efficient or even detrimental when the amount of lime is not adequately determined as referring to the site-specific requirements of the soil [7,8]. In order to reduce this risk of inefficient use of lime, the identification of soil management zones is generally recommended [9,10,11,12]. This is a relevant issue in Germany, where it is still common to apply the same amount of lime across the whole field [13]. This outdated approach implies that the lime is being homogeneously distributed on the sites, which of course, may suit soils with low heterogeneity of texture. However, it becomes crucial when the lime demand of the topsoil is spatially heterogeneous distributed, which is mostly the case [11,14,15,16,17,18,19,20,21,22,23]. In developing countries, liming is in many cases not feasible for economical and logistical reasons [24]. Therefore, site-specific approaches become meaningful since using lime must be done as efficiently as possible [24].

Site-specific lime demand can be determined using the spatial variation of both the soil apparent electrical conductivity (EC_a_) and the topsoil pH as input variables for lime demand prediction models [7,8,25,26]. The EC_a_ functions as offline data, i.e., data that only needs to be obtained once and is used for background information in the following years. This is possible due to the fact that the temporal variability of EC_a_ values is very low [27]. In contrast, the pH value is a temporally variable determinant [27] whose temporal changes must be taken into account when determining the lime demands. Thus, the topsoil pH needs to be evaluated immediately prior to any scheduled liming procedure. For both EC_a_ and pH, low-input determination approaches are available [8,28]. For EC_a_, it is the EM38 MK1 (Geonics Ltd., Mississauga, ON, Canada) [26,29,30,31], and for the pH it’s a flat electrode (FE) with a handheld pH meter (FieldScout pH 110 Meter, Spectrum-Technologies Ltd., Bridgend, UK). Soil EC_a_ mapping is a broad field of research with a long history [26,32,33,34,35,36,37]. Many different types of sensors for soil mapping have been developed including EM38 MK1, Veris 3100 (Veris Technologies Inc., Salina, CA, USA) and Geophilus Electricus [15,22,26,34,37,38,39]. The EM38 is one of the most promising [26,39,40,41,42], and has been thoroughly investigated in numerous studies [35,39,40,42]. It allows quick and low-cost soil EC_a_ mapping [36,42], wherein both the absolute values [43] and their spatial heterogeneity are reproducible.

Adequate correlations between EC_a_ data based on measurements with the EM38 MK1 and the soil texture characteristics have been reported [26,36,40,42,44,45]. Generally, four classes of texture (sand, loamy sand, loam and clay) can be detected using the EM38 MK1 (Table 1).

There are no relevant differences between EC_a_ readings from the EM38 MK1 and the subsequent model EM38 MK2 [39]. Considering the easy and quick use of the EM38 MK1 compared to direct EC measurement approaches such as Veris 3100 [15,26] and Geophilus Electricus [22], it has generated great interest among practitioners for determination of site-specific distributions of soil characteristics in the past two decades. The site-specific EC_a_ information allows for a better implementation of precision farming approaches such as tillage, sowing and fertilization [11]. However, there is only scarce information on both the EC_a_ measurement approach using the EM38 MK1 and the potential applicability of the EC_a_ data for describing lime fertilizer requirements on heterogeneous sites. Sanches et al. [7] showed that EC_a_ can represent the spatial distribution of pH values (average pH = 5.14) on a clayey site in Brazil. They found a high correlation of r = 0.59 between EC_a_ and soil pH [7]. Consequently, the EC_a_ values are likely sufficient for determining the lime demand under certain conditions (e.g., clayey soil [7]), because the pH is essential for estimating the current lime demand of a site [46]. However, Sanches et al. [7] suggested that EC_a_ data should not be used alone, but in combination with a site-specifically arranged (targeted) sampling mesh [7]. A similar approach is followed in Germany as well. Several large scale (field sizes of 50–200 ha) EC_a_ mappings across heterogeneous regions in northern Germany revealed that the EC_a_ does not correlate with the pH but rather the soil texture [47]. The soil texture is a fixed parameter (despite topsoil losses through erosion) upon which official recommendations for site-specific lime fertilization were calculated in Germany [46]. The EC_a_ can thus be used for generating offline maps showing the spatial distribution of the potential lime demand [42].

For estimating the current lime demand, the potential lime demand must be related to the current topsoil pH value. Therefore, an adequate non-destructive (low-cost) topsoil pH measurement must be taken whenever a lime application is planned [7]. In this context, several studies report on the Veris 3100—a device that combines EC_a_ and online pH measurement [8,15,48]. For this device, good correlations between in situ and ex situ determined topsoil pH values of arable soils were found [15,26,32]. However, this in situ sampling method is time consuming and still requires high-input and expensive equipment, despite being more efficient than destructive determination approaches for soil texture and pH [46,49]. Other low-input approaches, which are even less demanding than the Veris 3100 such as handheld pH-meters, may be relevant for agricultural production in developing countries—but as yet they have not been deeply investigated.

The aim of this study was to gain more insights to the potential suitability of a low-input in situ pH-meter for low-input determination of the site-specific lime demand of arable fields. We hypothesized that in situ determination based on a handheld pH meter results in similar pH values to the reference (ex situ) approach and that it is therefore possible to determine the lime demand using the low-input approach.

## 2. Materials and Methods

To fulfil the aim of this study, a field study was conducted in spring 2012. In the following sections it will be described (i) which sites were chosen for investigation and when the field study was conducted, (ii) how EC_a_ was measured, (iii) which pH-measurements were applied, (iv) how the lime demand was calculated, and (v) how both statistical analysis and the overall performance evaluation were conducted.

### 2.1. Description of Experimental Sites

**Figure 1:**
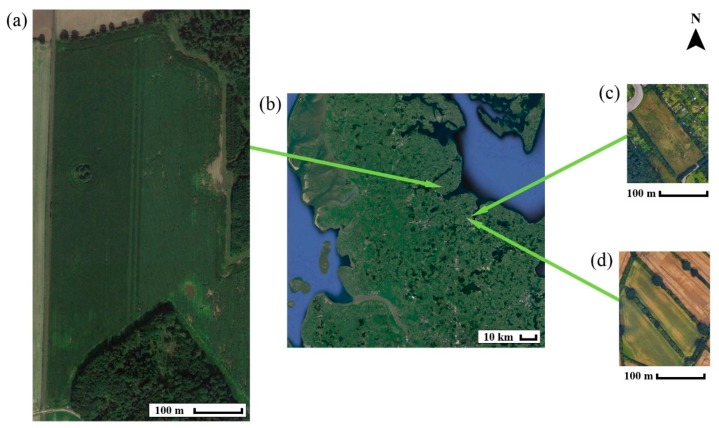
Overview of shape, size and location of the sampling sites ‘Hochwollhagen’ (**a**), ‘Bremerskamp’ (**c**) and ‘Suchsdorf’ (**d**). The green arrows point at the locations of the sites in Schleswig-Holstein (**b**) (north Germany). The pictures were adapted from Google Earth Pro (© 2018 Google LLC).

In this sub-section, some details are provided on the sampling-sites. In total, three sampling sites were selected for this study due to their expected low pH values. The first site (‘Hochwollhagen’) belongs to a dairy cattle farm with 500 ha of cultivation area located near Eckernfoerde in North-Germany (54°30′57.4″ N, 9°49′04.6″ E, 24 m above sea level) (Figure 1a). The field Hochwollhagen has a size of 23.4 ha, and it is surrounded by farm tracks to the north and the west, and by woodlands to the south and the east (Figure 1a). In the southeast, the field is connected to another field as shown in Figure 1a. The field was selected according to the results of prior research studies with EM38, which indicated that the texture of the site was both highly varied and heterogeneously distributed. The second restriction for field selection was that for several years prior to the measurements in 2012 no liming should have occurred, which was the case at Hochwollhagen. The other two sites ‘Bremerskamp’ (54°20′56.6″ N, 10°06′26.2″ E, 24 m above sea level) and ‘Suchsdorf’ (54°21′02.9″ N, 10°04′05.1″ E, 12 m above sea level) are comparatively small (<2 ha) and located near Kiel (Figure 1, Table 2). The measurements of both EC_a_ and in-situ pH were conducted in spring 2012.

### 2.2. The Determination of Soil Apparent Electrical Conductivity

**Figure 2:**
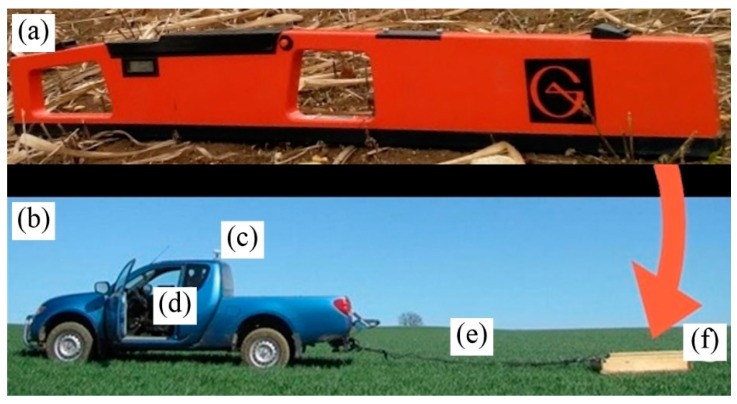
Experimental setup for EC_a_ measurements using the EM38 MK1 (**a**): Pickup truck (**b**) with GPS-module (**c**) and DASY-Lab (**d**) pulling a wooden sledge (**f**) on a 4.5 m long rope (**e**). The cables for data transfer and power supply were attached to the rope via cable ties. The EM38 MK1 (A) was placed in vertical mode within a wooden box on the sledge covered in foam. Where necessary, plastic screws and tensioning ropes were used to increase stability of the sledge.

The EM38 MK1 (Figure 2a) was fixed to a wooden sledge (Figure 2f). The sledge was pulled by a pickup truck (Figure 2b) driving at on average 15 km h^−1^.

The distance between the tracks was set to 10 m ± 1 m accuracy depending on coastal GPS-signal. The whole pathway was both monitored online and recorded using DASY-Lab (measX GmbH & Co. KG, Mönchengladbach, Germany) GPS module (CSI Inc., Wheaton, IL, USA).

The GPS-data was merged with EM38-data and recorded in 1 s intervals. The GPS-antenna was 7 m away from the middle of the sledge, because the sledge was pulled 4.5 m behind the pickup truck (Figure 2e,f) and the distance from the antenna (Figure 2c) to the back of the pickup truck measured 2.5 m. Therefore, an offset of the GPS-signal of 7 m was calculated within DASY-Lab when merging GPS and EM38 data streams. The DASY-Lab module was set to calculate average EC_a_ values for each GPS recording interval (1 s). This was necessary since prior tests have shown that average EC_a_ values ensure a better representativeness for the EC_a_ than single EC_a_ values.

The power supply for EM38 was provided via cable connection to the car battery to avoid any voltage fluctuations as has been found to be relevant when using a 9 V-battery (as recommended by Geonics) in prior tests. EM38 measurements were conducted from 8.00 AM to 9.00 AM after thoroughly calibrating the EM38 as described within the EM38-Guidelines provided by Geonics. Additionally, the EC_a_ values of the starting point were measured once again after the whole field was measured. EC_a_ mapping was immediately done afterwards using ARC View (ESRI Inc., Redlands, CA, USA) to check whether the EC_a_ data was complete and correct, e.g., if there were any shifts in absolute EC_a_ values during the measurements via comparison of EC_a_ values at the starting point at the start and at the end of EM38 measurement. To illustrate the estimated spatial distribution of soil texture, EM38 data were assigned to the classification of Domsch and Giebel [40] (Table 1).

### 2.3. In Situ and Ex Situ Determination of Topsoil pH

The pH value of each soil sample was determined both directly in the field (in situ) and according to standard laboratory practice (ex situ). Both methods are detailed in the following subchapters.

#### 2.3.1. Soil Sampling and In Situ Determination of Topsoil pH

**Figure 3:**
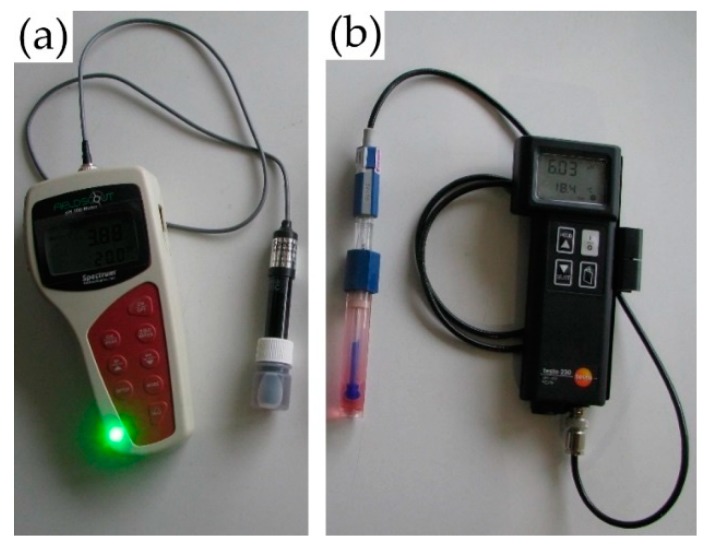
Electrodes and pH meters used for in situ (FieldScout pH 110 Meter, Spectrum-Technologies Ltd., Bridgend, UK) (**a**) and ex situ (testo230, Testo SE & Co. KGaA, Titisee-Neustadt, Germany) (**b**) determination of topsoil pH.

For the collection of soil samples, a sampling grid was created using ARC View following the EC_a_ measurement. In this way, each soil sampling point was assigned to an EC_a_ sampling point with an accuracy of ±1 m (coastal GPS-signal). The soil sampling points of this sampling grid were then individually visited to take topsoil samples. At each soil sampling point, topsoil samples were taken from a depth of 0–30 cm using a boring rod of 18 mm diameter. Right after the rod was pulled out, 5 individual in situ pH measurements were taken on each soil sample using a handheld pH-meter in combination with a FE (Figure 3a). The measurements were carried out at regular intervals (5, 10, 15, 20, 25 cm depth) directly on the earth still in the drill rod.

For each individual measurement, attention was paid to measurements of equal length and pressure. After each measurement, the FE was cleaned within distilled water (H_2_O_DEST_) and dried with a clean part of a kitchen towel. Note that the FE was dabbed and not rubbed with the kitchen towel, as friction turned out to be sub-optimal for calibration stability. After ten measurements, a two-point calibration was carried out (pH 4 and pH 7) according to the manufacturer’s specifications (Spectrum-Technologies Ltd., Bridgend, UK). To avoid any shifts between the measurements, the last probe before re-calibration was re-measured after re-calibration once again as control. Prior to the statistical analysis, the average in-situ pH values were calculated. After each measurement of a soil sample was completed, the soil sample was poured into a cup, roughly homogenized, transferred to an aluminum dish and weighed together with the aluminum dish (FM weight). This FM-value was later used to calculate the water content of the soil sample. The samples were stored in a cool box at 5–8 °C until further processing.

#### 2.3.2. Ex-Situ Determination of Topsoil pH

In the laboratory, the samples were dried to constant weight, weighed back (DM weight) and coarsely ground with a mill (Retsch GmbH, Haan, Germany). Further sample preparation and pH measurement were carried out according to VDLUFA specifications [49] using a standard ex situ pH-probe (testo230, Testo SE & Co. KGaA, Titisee-Neustadt, Germany). Additionally, the ex situ approach was conducted twice: (i) ‘pH_REFCaCl_’ using CaCl_2_ [39] and ‘pH_REFH2O_’ using H_2_O_DEST_ instead of CaCl_2_ to examine the effect of CaCl_2_.

### 2.4. Statistical Analysis

A multiple linear regression (MLR) for the soil properties and the potential lime demand was developed using the PROG REG procedure of SAS ^®^Proprietary Software 9.4 TS level 1M5 (SAS Institute Inc., Cary, NC, USA) based on recommendations by VDLUFA [46] (Figure A1). Both linear and non-linear regressors as well as an intercept were put into the model. For the selection of regressors, stepwise selection was used. A correlation matrix was generated using the PROC CORR procedure of SAS ^®^Proprietary Software 9.4 TS level 1M5.

### 2.5. Lime Demand Calculation

The lime demand was calculated according to official recommendations for determining the lime demand of arable and grassland soils based on the pH value [46]. Therefore, the abovementioned MLR was used to consider both EC_a_ and pH when calculating the specific lime demand for each observation. For the presentation of the interpolated spatial distribution of the lime demands, ARC View (ESRI Inc., Redlands, CA, USA) was used.

## 3. Results & Discussion

### 3.1. EC_a_ and pH Distribution

**Figure 4:**
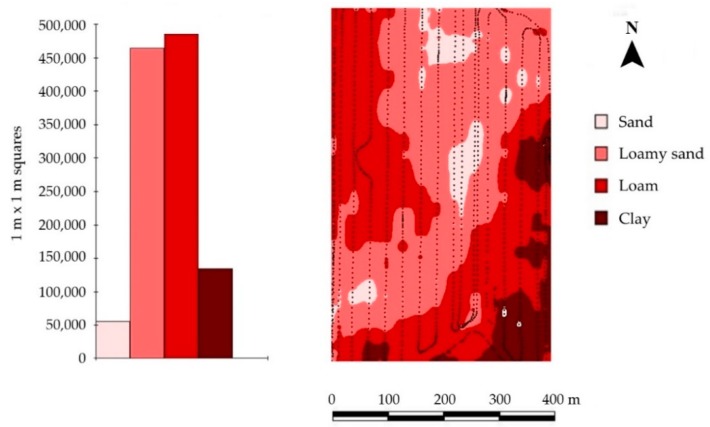
Histogram and spatial distribution of estimated soil texture (according to Domsch and Giebel [29] and Reckleben and Lamb [31]) based on EC_a_ determination using the EM38 MK1 (Geonics, Canada) in vertical mode at the site Hochwollhagen (north Germany).

**Figure A2:**
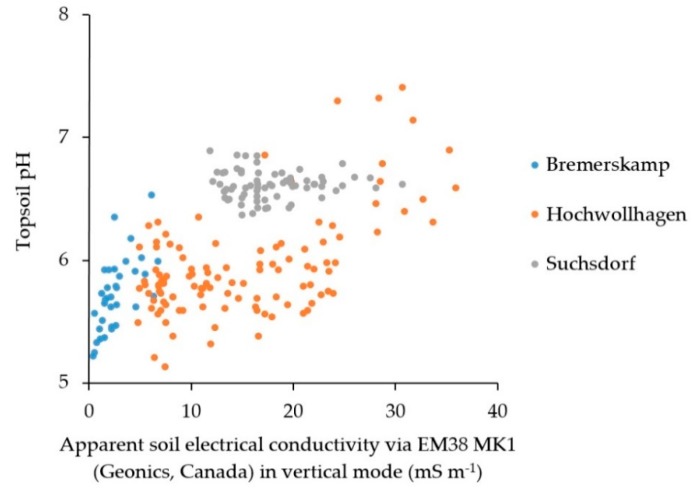
Scatterplot of ex situ determined topsoil pH and EC_a_ (via EM38 in vertical mode) of the three experimental sites used in this study (n = 214).

All sites (Hochwollhagen, Bremerskamp and Suchsdorf) showed a heterogeneous spatial distribution of both EC_a_ and pH-values (Figure 4 and Figure A2; Table 3). This heterogeneous spatial distribution of EC_a_ implies within-field variability of crop growth [50]. Therefore, it is necessary to plan site-specific management [11,51,52]. The average topsoil pH of the sites ranged from 5.72 (Bremerskamp) to 6.61 (Suchsdorf) (Table 3) (Figure A2). The average EC_a_ values of the sites ranged from 2.6 mS m^−1^ (Bremerskamp) to 17.9 mS m^−1^ (Suchsdorf) (Table 3) (Figure A2). Sandy areas (<5 mS m^−1^) were only observed on the sites Hochwollhagen and Bremerskamp, following the recommendations by Domsch and Giebel [40] and Reckleben and Lamb [42] for texture-related EM38 classification (Table 1). However, the distribution of EC_a_ values was higher at both Hochwollhagen and Suchsdorf compared with Bremerskamp (Table 3). Therefore, the results of Hochwollhagen have been used to illustrate the comparison between in situ topsoil pH- and ex situ topsoil pH-based lime demands. However, data from all three sites were used for the calculations of the correlations between in situ and ex situ determined topsoil pH.

**Figure 5:**
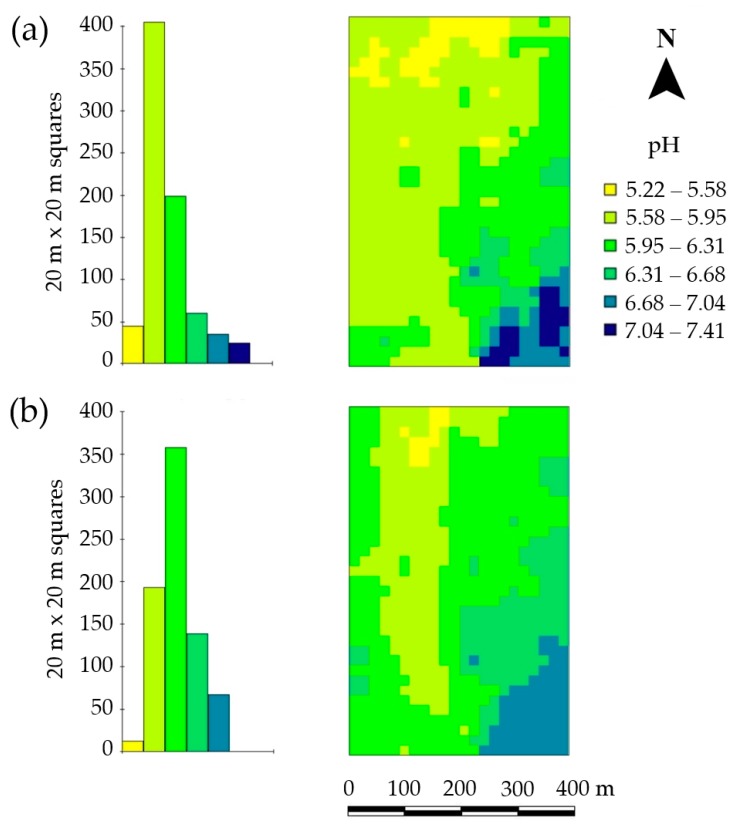
Histogram and spatial distribution of ex situ (**a**) and in situ (**b**) determined topsoil pH at site Hochwollhagen (north Germany). Note, that the same color scale was used for both maps (**a**,**b**).

A comparison of the spatial distribution of in situ and ex situ determined topsoil pH values revealed that the in situ pH determination produces a map that is basically comparable with the ex situ pH determination (Figure 5). Overall, this is in line with literature data [8]. However, there were clear differences in the very low (5.22–5.58) and the very high range of pH values (7.04–7.41) (Figure 5).

### 3.2. The Correlation Between EC_a_ and Ex Situ Determined pH—Implications for Lime Demand Estimation

**Figure 6:**
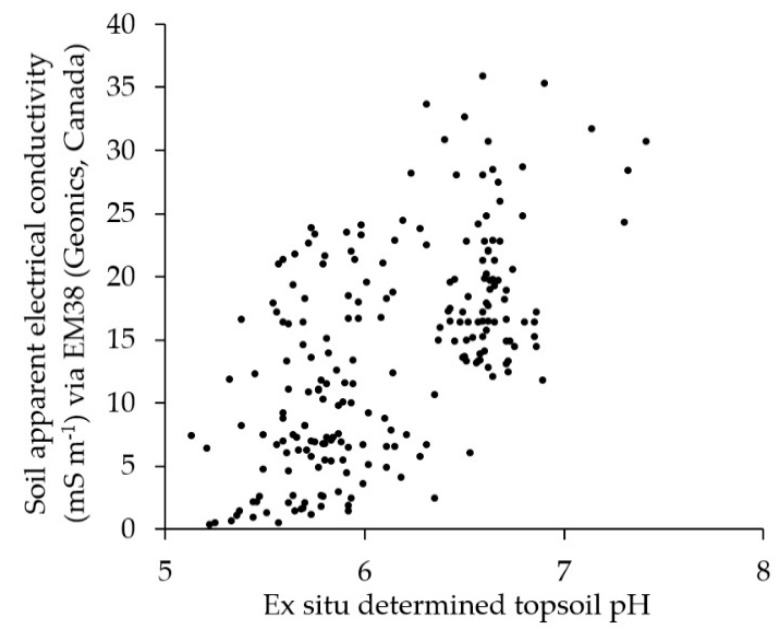
Scatter plot of the correlation between EC_a_ (via EM38 vertical, Geonics, Canada) and the ex situ determined topsoil pH (n = 214).

The EC_a_ significantly correlated with the ex situ determined pH (Figure 6, Table 4), whereas only about 60% (r = 0.596) of the pH values could be explained using EC_a_. These values correspond well with those of Mahmood et al. [53] and Cambouris et al. [54], who found an average correlation between ECa and pH of r = 0.513 and r = 0.449, respectively. Consequently, EC_a_ would not have been sufficient to explain the spatial variation in soil. This was in line with the findings from other studies in which similar methods were employed [26,47]. Consequently, it was expected that the EC_a_ does not explain the estimated lime demand (LD_e_). The LD_e_ was calculated from EC_a_ and pH according to recommendations by VDLUFA (Figure A1). However, the EC_a_ was necessary for taking the spatial variation of soil texture-related potential lime demand (LD_p_) into account, because following VDLUFA [46], different soils have different lime demands (Figure A1). Furthermore, there was only a low correlation between the EC_a_ and the topsoil humidity (r = 0.256). This value was much lower compared with the findings by Sun et al. [11] who reported a correlation between EC_a_ and volumetric soil water content of r = 0.663 [11]. Sudduth et al. [55] also reported a higher correlation between EC_a_ and soil moisture (r = 60.4). This is probably due to the fact that at the time of the measurement of the present study the soil was less saturated than in the studies by Sun et al. [11] and Sudduth et al. [55]. The reason for this is that under drier conditions the influence of the soil water content on the EC_a_ is superimposed by the stronger influence of the texture [30].

Heil and Schmidhalter [26,30] proposed to use the EC_a_ values not in absolute terms but as a covariate, because the absolute EC_a_ values strongly depend on soil and weather conditions [30]. Against this, other studies have shown good correlations between EC_a_ (EM38) and the soil texture [40,42] as far as both similar surrounding conditions and calibration methods are used. Field observations conducted prior to those presented in this study have outlined the importance of a continuous power supply without any current fluctuations during the measurement. In this study, both similar surrounding conditions (including thorough calibrations) and continuous power supply for the EM38 were given. Thus, it was decided to use the absolute EC_a_ values of the EM38 to represent the soil texture (Table 1) and use this information as covariate for calculating the site-specific LD_e_ (Figure A1). Thus, the current pH information is also required for estimating the lime demand as was also reported by Lund et al. [8].

### 3.3. The Correlation Between In-Situ and Ex-Situ Determined Topsoil pH

A strong correlation between in situ and ex situ determined topsoil pH was found (r = 0.888, *p* < 0.0001) (Table 4) which is in line with the literature [8,36]. Lund et al. [8] also found an almost identical correlation between in situ (on-the-go) and ex situ (manually) determined pH of r = 0.894 [8]. In the present study however, the correlation was further improved through non-linear regression (r = 0.924–0.928, *p* < 0.0001), whereas the extended model (Model 1) only marginally improved the model accuracy (Table 5). This was also expected because soil humidity and topsoil pH did not correlate (Table 4). Here, less complex models are preferred in terms of interpretability and practical implementation [56]. Therefore, the less complex model (Model 2) (Table 5) was used for the comparison of ex situ (pH_REF_), in situ (pH_FE_) and modified in situ (pH_FEM1_) pH-based lime demands.

### 3.4. Effects of In Situ and Ex Situ Determined Topsoil pH on the Estimated Lime Demand Distribution

**Figure 7:**
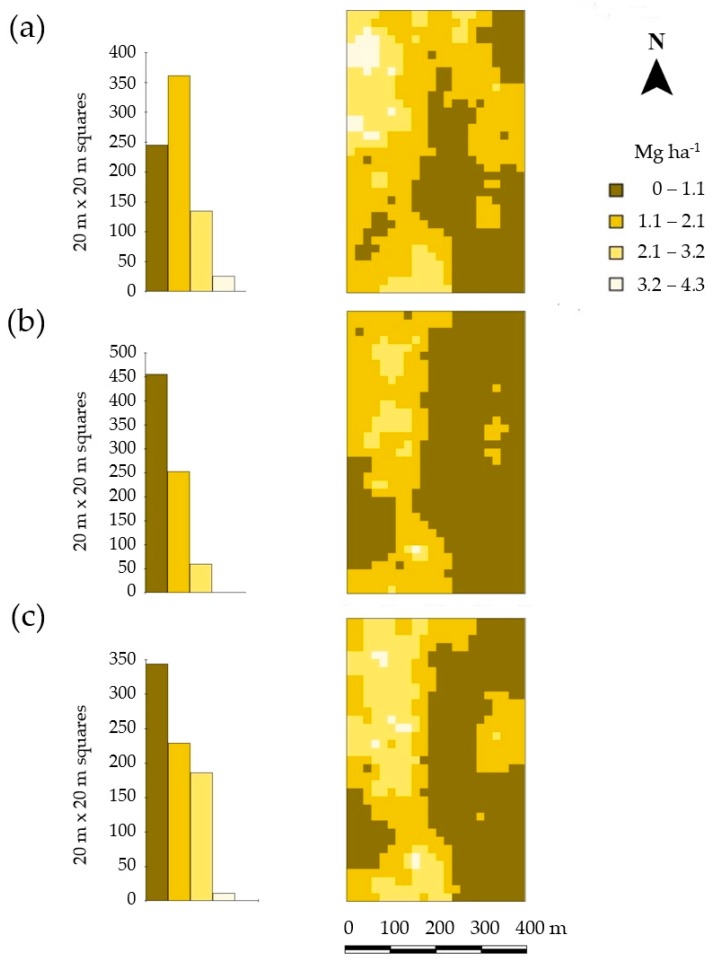
Spatial distribution of estimated lime (CaO) demand at site Hochwollhagen based on ex situ determined pH (**a**), in situ determined pH (**b**), and modified in situ determined pH (**c**). For (**c**) a simple non-linear model (M2) was used. The regressors included in M2 are the in situ determined pH, the EC_a_, and all two-fold interactions between them.

**Figure A3:**
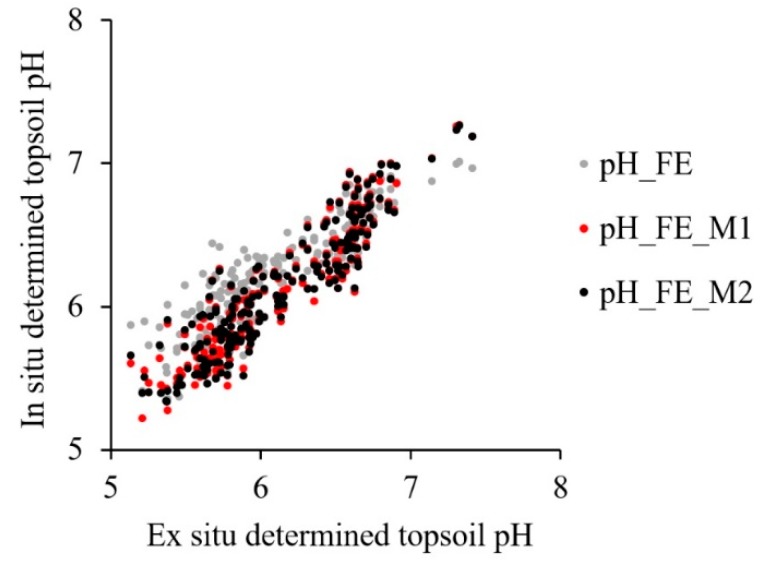
Scatterplot of in-situ (pH_FE = pH measured using a flat electrode attached to a handheld pH meter) and ex-situ pH values. For in-situ pH, both observed and modified values (M1, Model 1; M2, Model 2) are shown (n = 214).

The range of variation in lime demand was higher at the site Hochwollhagen (−0.62–4.18 Mg ha^−1^) than at the sites Bremerskamp (−1.35–0.67 Mg ha^−1^) and Suchsdorf (−0.64–1.59 Mg ha^−1^) (Table S1). This was expected due to a higher soil heterogeneity (Table 3). The average lime demand of Hochwollhagen amounted to 1.6 ± 1.2 Mg ha^−1^, whereby the average lime demand in the western part of the field was found to be almost twice as high as in the eastern part (Figure 7). This underlines the importance of site-specific lime management: If lime had been applied uniformly across the entire field according to the calculated demand, almost half of the field would have received an excess of about 1 Mg CaO ha^−1^, while the rest of the field would have received about 1 Mg CaO ha^−1^ less than required (Table S1). Using EC_a−_ and pH-measurements it would be possible to quantify the heterogeneity of lime demands and to proceed accordingly (through site-specific lime management) (Figure 7), as was also suggested by Sanches et al. [7]. However, the in situ determination of topsoil pH would result in a rather restrained lime management (Figure 7b). This is because the subareas with high lime demand are rather underestimated. Here, a slight modification of the in situ pH values (M2) helps further improve the in situ-based lime demand estimate. The subareas with higher lime demands are much better represented in comparison with the unmodified in situ pH values (Figure 7c). This is mainly due to a better representation of pH values below six (Figure A3). This also fits well with the results of Sanches et al. [7], as in their study the average pH value of the soil was also below six [7].

Overall, the results indicate, that the use of a handheld in situ pH-meter can be effectively used for topsoil pH determination on comparable sites - as far as accuracy is concerned. In this study, the comparison of in situ and ex situ determined pH values revealed, that the accuracy of comparably cheap (low-input) in situ measurements is only slightly lower compared with ex situ measurements, given a pH ranging from 5.13 to 7.41.

The results of Gebbers et al. [57] refer to a smaller area compared with the fields used in this study (Table 1). Also, the EC_a_ was measured with a less suitable method for upscaling processes compared with the EM38 [15,25,58]. This is because (i) the Veris 3100 is pulled much more slowly than the EM38, and (ii) the equipment is much heavier and therefore, less easy to handle than the EM38 [26] (Figure 3). In the present study, the in situ measurement of the pH was taken within topsoil. This is more representative than the measurement conducted by Schirrmann et al. [25] who reported inaccuracies in pH measurement because of plant residual material and other reasons. This explains why the correlation between ex situ- and in situ determined pH was found to be higher in the present study compared with Schirrmann et al. [25].

### 3.5. Consequences for Practical Implementation and Future Research Directions

The results indicate that the low-input approach of current lime demand determination presented here is ready for large scale implementation. This is because, low-input measurements need to be fast, cheap and easy to understand while simultaneously being accurate enough to allow for successful precision farming applications such as site-specific liming. These requirements are met by the methodological approaches of both EM38-based EC_a_ measurement and in situ determination of topsoil pH using a handheld pH-meter. Regarding the EC_a_ measurement, these results are in line with findings from Heil et al. [26] and Koganti et al. [15]. Further research should focus on how the methodological approach presented here could contribute to the implementation of site-specific lime management in less developed countries, for example what a cost-benefit calculation would look like, or what teaching effort would be necessary to enable a correct application of the method. Therefore, future research on this topic may help to improve both environmental and economic performance of agricultural production on heterogeneous, specifically marginal agricultural lands [58,59].

## 4. Conclusions

Within humid climate zones, the agricultural land requires lime application to maintain fertile and productive. In this study, the current lime demand of three experimental sites in northern Germany was found to be heterogeneously distributed, which was to be expected, as the soil properties determining the lime demand are usually heterogeneous. Therefore, the approach of homogeneous lime application was unsatisfactory in terms of good agricultural practices. This study shows that it is possible to determine the current lime demand using low-input approaches. Here, the combined use of EC_a_ (via EM38) and in situ determined topsoil pH (via handheld pH-meter) was found to be suitable for low-input lime demand determination.

## Figures and Tables

**Table 1:** Available information on the interpretability of the EC_a_ values assessed via EM38 MK 1 in terms of soil texture.

Texture	Domsch and Giebel [40]	Reckleben and Lamb [42]
	EC_a_ Class (mS m^−1^)	
Sand	1–6	1–8
Loamy sand	5–16	8–16
Loam	16–36	16–35
Clay	30–96	35–70

**Table 2:** Overview of soil properties of the three experimental sites (Hochwollhagen, Bremerskamp and Suchsdorf) chosen for this study.

Name	Size (ha)	Soil Properties	P (mg 100 g^−1^)	K (mg 100 g^−1^)	Mg (mg 100 g^−1^)	Date of EM38-Measurement
Hochwollhagen	23.4	Sandy loam	6.1	12.5	5.7	22.02.2012
Bremerskamp	0.8	Sand	10.5	14.1	6.6	15.02.2012
Suchsdorf	1.3	Sandy loam	6.6	15.8	4.2	08.03.2012

**Table 3:** Overview of observations for EC_a_, ex situ determined topsoil pH and topsoil humidity at the three experimental sites (Hochwollhagen, Bremerskamp and Suchsdorf).

Site	N	Minimum	Average	Median	Maximum
		**EC_a_ (mS m^−1^)**			
Hochwollhagen	110	4.8	15.1	13.0	35.9
Bremerskamp	35	0.4	2.6	2.2	6.7
Suchsdorf	69	11.8	17.9	16.5	30.7
		**Topsoil pH**			
Hochwollhagen	110	5.13	5.95	5.84	7.41
Bremerskamp	35	5.22	5.72	5.70	6.53
Suchsdorf	69	6.37	6.61	6.61	6.89
		**Topsoil humidity (wt.%)**			
Hochwollhagen	110	9.0	14.4	14.0	25.0
Bremerskamp	35	20.0	21.9	22.0	24.0
Suchsdorf	69	11.0	14.7	15.0	17.0

**Table 4:** Correlation (r) matrix for the parameters (i) apparent soil electrical conductivity (EC_a_), (ii) soil humidity (SH), (iii) ex situ determined topsoil pH (pH_REF_), (iv) in situ determined topsoil pH (pH_FE_), and (v-vi) two modified versions of pH_FE_ using Model 1 (pH_FEM1_) and Model 2 (pH_FEM2_) (*** = *p* < 0.0001; ** = *p* < 0.01; * = *p* < 0.05; n.s. = not significant) (n = 214).

	SH	pH_REF_	pH_FE_	pH_FEM1_	pH_FEM2_
EC_a_	−0.259 **	0.596 ***	0.475 ***	0.643 ***	0.646 ***
SH		−0.098 n.s.	−0.047 n.s.	−0.106 n.s.	−0.143 *
pH_REF_			0.888 ***	0.928 ***	0.924 ***
pH_FE_				0.957 ***	0.961 ***
pH_FEM1_					0.996 ***

**Table 5:** Models for prediction of ex situ determined topsoil pH using the input variables in situ determined topsoil pH (pH_FE_), soil apparent electrical conductivity (EC_a_), soil humidity (not in Model 2), and all two-fold interactions, including the squared terms. A stepwise selection was chosen to select relevant regressors (significance level: *p* < 0.15) (*** = *p* < 0.0001; ** = *p* < 0.01; * = *p* < 0.05).

Intercept/Regressor	Model 1 (r = 0.9276 ***)			Model 2 (r = 0.9237 ***)		
	Estimate	Standard Error	*p*-Value	Estimate	Standard Error	*p*-Value
Intercept	12.8995	3.2877	0.0001	15.4019	3.2370	<0.0001
pH_FE_	−3.7371	1.0371	0.0004	−4.0646	1.0474	0.0001
EC_a_ × pH_FE_	0.0053	0.0013	0.0001	0.0049	0.0010	<0.0001
pH_FE_ × pH_FE_	0.4007	0.0841	<0.0001	0.4045	0.0847	<0.0001
EC_a_ × EC_a_	−0.0007	0.0002	0.0049	−0.0006	0.0002	0.0006
Soil humidity	0.1889	0.0684	0.0062			
Soil humidity × Soil humidity	−0.0019	0.0013	0.1307			
Soil humidity × pH_FE_	−0.0193	0.0114	0.0913			

## Supplementary Materials

The following are available online at https://www.mdpi.com/1424-8220/19/23/5280/s1, Table S1. Overview of combined observations for soil apparent electrical conductivity (via EM38 MK1 (Geonics, Mississauga, Canada) in vertical mode), soil humidity, ex situ determined topsoil pH (pH_REF_), in situ determined topsoil pH (pH_FE_), modified in situ determined topsoil pH (pH_FEM2_) and the respective estimated lime demands based on EC_a_ and pH (pH_REF_, pH_FE_, pH_FEM2_) values.
