# Supplementary material for: Low-Input Estimation of Site-Specific Lime Demand Based on Apparent Soil Electrical Conductivity and In Situ Determined Topsoil pH
Source: Sensors (Basel). 2019 Nov 30;19(23):5280. doi: 10.3390/s19235280 (PMC6928849; doi:10.3390/s19235280)
Supplement: Supplementary file 1 [file sensors-19-05280-s001.pdf]

Supplementary Information

Low-Input Estimation of Site-Specific Lime Demand Based on Apparent Soil Electrical Conductivity and In Situ Determined Topsoil pH

Moritz von Cossel [1,*], Harm Druecker [2] and Eberhard Hartung [2,*]

[1] Department of Biobased Products and Energy Crops (340b), Institute of Crop Science, University of Hohenheim, Fruwirthstr. 23, Stuttgart, 70599, Germany

[2] Institute of Agricultural Engineering, Kiel University, Olshausenstr. 40, Kiel, 24098, Germany

* Correspondence: moritz.cossel@uni-hohenheim.de (M.V.C.); ehartung@ilv.uni-kiel.de (E.H.); Tel.: +49-711-459-23557 (M.V.C.); +49-431-880-2107 (E.H.)

Table S1. Overview of combined observations for soil apparent electrical conductivity (via EM38 MK1 (Geonics, Mississauga, Canada) in vertical mode), soil humidity, ex situ determined topsoil pH (pH_{REF}), in situ determined topsoil pH (pH_{FE}), modified in situ determined topsoil pH (pH_{FEM2}) and the respective estimated lime demands based on EC_a and pH (pH_{REF}, pH_{FE}, pH_{FEM2}) values.

Site	EC_a	Soil humidity	pH_{REF}	pH_{FE}	pH_{FEM2}	$Lime_{REF}$	$Lime_{FE}$	$Lime_{FEM2}$
	mS m^{-1}	wt.%	-	-	-		Mg CaO ha^{-1}	
Bremerskamp	2.5	24	6.35	6.48	6.13	-1.35	-1.50	-1.02
Bremerskamp	6.1	22	6.53	6.66	6.45	-0.94	-1.11	-0.83
Bremerskamp	1.5	22	5.92	6.19	5.79	-0.89	-1.33	-0.64
Bremerskamp	1.9	21	5.92	6.07	5.69	-0.80	-1.06	-0.34
Bremerskamp	4.1	24	6.18	6.52	6.22	-0.78	-1.27	-0.84
Bremerskamp	2.5	21	5.93	6.20	5.82	-0.68	-1.14	-0.47
Bremerskamp	1.2	22	5.73	5.91	5.54	-0.60	-0.94	-0.19
Bremerskamp	1.8	21	5.78	6.10	5.71	-0.55	-1.13	-0.41
Bremerskamp	3.6	22	5.99	6.30	5.95	-0.55	-1.07	-0.48
Bremerskamp	3.0	21	5.87	6.13	5.77	-0.45	-0.92	-0.25
Bremerskamp	0.5	22	5.57	5.92	5.53	-0.44	-1.13	-0.35
Bremerskamp	1.6	20	5.68	6.08	5.69	-0.39	-1.14	-0.41
Bremerskamp	1.7	22	5.69	6.22	5.82	-0.39	-1.33	-0.66
Bremerskamp	2.6	22	5.79	6.12	5.75	-0.38	-0.99	-0.30
Bremerskamp	1.5	21	5.65	5.98	5.60	-0.35	-1.00	-0.25
Bremerskamp	2.7	22	5.78	6.02	5.67	-0.34	-0.80	-0.09
Bremerskamp	2.1	22	5.70	5.81	5.50	-0.31	-0.54	0.15
Bremerskamp	5.1	22	6.02	6.24	5.93	-0.29	-0.69	-0.10
Bremerskamp	4.5	22	5.91	6.34	6.02	-0.19	-0.96	-0.41
Bremerskamp	2.1	23	5.62	5.84	5.52	-0.13	-0.60	0.10
Bremerskamp	1.3	23	5.51	5.94	5.57	-0.09	-0.97	-0.22
Bremerskamp	2.7	20	5.64	5.73	5.47	-0.03	-0.23	0.40
Bremerskamp	1.0	22	5.44	5.69	5.40	0.00	-0.57	0.10
Bremerskamp	5.5	24	5.89	6.40	6.11	0.07	-0.86	-0.38
Bremerskamp	6.7	22	5.99	6.17	5.90	0.11	-0.26	0.31
Bremerskamp	0.7	23	5.33	5.71	5.40	0.19	-0.68	0.01

Site	EC_a	Soil humidity	pH_{REF}	pH_{FE}	pH_{FEM2}	$Lime_{REF}$	$Lime_{FE}$	$Lime_{FEM2}$
	$mS\ m^{-1}$	wt.%	-	-	-		$Mg\ CaO\ ha^{-1}$	
Bremerskamp	1.1	21	5.36	5.58	5.35	0.23	-0.30	0.26
Bremerskamp	2.2	22	5.46	5.81	5.50	0.28	-0.52	0.17
Bremerskamp	1.5	22	5.37	5.54	5.34	0.31	-0.11	0.39
Bremerskamp	2.2	22	5.44	5.71	5.44	0.33	-0.31	0.32
Bremerskamp	0.5	21	5.25	5.73	5.41	0.34	-0.77	-0.06
Bremerskamp	2.6	22	5.47	5.72	5.46	0.36	-0.23	0.39
Bremerskamp	0.4	21	5.22	5.90	5.51	0.39	-1.12	-0.34
Bremerskamp	4.6	21	5.62	5.91	5.63	0.49	-0.17	0.47
Bremerskamp	6.3	21	5.71	5.84	5.62	0.67	0.36	0.91
Hochwollhagen	5.8	12	6.28	6.47	6.20	-0.62	-0.91	-0.49
Hochwollhagen	30.7	17	7.41	6.97	7.19	-0.56	0.53	-0.04
Hochwollhagen	6.7	15	6.31	6.61	6.40	-0.51	-0.95	-0.65
Hochwollhagen	4.9	11	6.11	6.35	6.04	-0.50	-0.90	-0.37
Hochwollhagen	24.3	14	7.30	7.00	7.24	-0.41	0.19	-0.30
Hochwollhagen	28.4	14	7.32	7.01	7.26	-0.37	0.35	-0.25
Hochwollhagen	6.6	11	6.15	6.34	6.07	-0.24	-0.58	-0.08
Hochwollhagen	7.5	18	6.21	6.41	6.17	-0.18	-0.53	-0.10
Hochwollhagen	6.6	12	6.11	6.28	6.01	-0.16	-0.47	0.05
Hochwollhagen	17.2	18	6.86	6.82	6.89	-0.05	0.03	-0.10
Hochwollhagen	7.9	13	6.13	6.17	5.92	0.05	-0.03	0.50
Hochwollhagen	31.7	14	7.14	6.88	7.03	0.09	0.83	0.39
Hochwollhagen	10.7	16	6.35	6.46	6.29	0.10	-0.11	0.21
Hochwollhagen	5.4	11	5.83	6.10	5.80	0.18	-0.38	0.24
Hochwollhagen	4.9	13	5.77	6.02	5.72	0.20	-0.33	0.31
Hochwollhagen	6.5	11	5.92	6.23	5.95	0.22	-0.40	0.15
Hochwollhagen	5.5	12	5.80	6.16	5.86	0.27	-0.47	0.13
Hochwollhagen	8.8	13	6.10	6.31	6.09	0.29	-0.14	0.32
Hochwollhagen	6.9	17	5.88	5.66	5.52	0.40	0.94	1.33
Hochwollhagen	5.8	10	5.73	6.06	5.78	0.51	-0.22	0.39
Hochwollhagen	9.2	13	6.02	6.34	6.13	0.54	-0.13	0.30
Hochwollhagen	7.1	18	5.83	6.00	5.76	0.56	0.17	0.74
Hochwollhagen	6.8	15	5.80	5.96	5.72	0.56	0.19	0.77
Hochwollhagen	7.6	13	5.87	6.10	5.85	0.57	0.06	0.61
Hochwollhagen	7.3	13	5.84	6.31	6.05	0.58	-0.40	0.09
Hochwollhagen	6.8	16	5.79	6.02	5.77	0.59	0.07	0.65
Hochwollhagen	7.3	12	5.81	6.26	6.00	0.65	-0.31	0.21
Hochwollhagen	19.8	16	6.64	6.38	6.31	0.66	1.32	1.50
Hochwollhagen	6.9	14	5.75	6.16	5.89	0.71	-0.20	0.37
Hochwollhagen	6.3	15	5.67	6.44	6.18	0.77	-0.78	-0.35
Hochwollhagen	7.0	12	5.73	6.11	5.85	0.78	-0.08	0.49
Hochwollhagen	12.4	17	6.14	6.24	6.07	0.84	0.61	1.00
Hochwollhagen	35.3	23	6.90	6.87	6.98	0.86	0.96	0.59

Site	EC_a	Soil humidity	pH_{REF}	pH_{FE}	pH_{FEM2}	$Lime_{REF}$	$Lime_{FE}$	$Lime_{FEM2}$
	$mS\ m^{-1}$	wt.%	-	-	-		$Mg\ CaO\ ha^{-1}$	
Hochwollhagen	4.8	13	5.49	6.15	5.84	0.88	-0.59	0.03
Hochwollhagen	6.1	13	5.61	6.23	5.94	0.88	-0.48	0.08
Hochwollhagen	10.0	15	5.93	6.22	6.01	0.92	0.25	0.71
Hochwollhagen	28.7	21	6.79	6.81	6.93	0.96	0.90	0.57
Hochwollhagen	9.8	13	5.87	6.16	5.95	1.03	0.34	0.82
Hochwollhagen	7.3	11	5.66	6.19	5.93	1.03	-0.18	0.36
Hochwollhagen	10.1	14	5.89	6.17	5.97	1.04	0.37	0.85
Hochwollhagen	8.2	15	5.70	5.57	5.50	1.13	1.49	1.71
Hochwollhagen	8.2	9	5.70	6.20	5.96	1.13	-0.03	0.48
Hochwollhagen	7.5	16	5.64	5.72	5.56	1.13	0.92	1.34
Hochwollhagen	7.0	14	5.59	5.99	5.75	1.15	0.17	0.74
Hochwollhagen	6.7	12	5.56	5.68	5.53	1.16	0.84	1.26
Hochwollhagen	11.5	15	5.94	6.25	6.07	1.18	0.44	0.85
Hochwollhagen	11.6	13	5.90	6.05	5.88	1.31	0.92	1.36
Hochwollhagen	10.3	16	5.79	5.69	5.59	1.34	1.62	1.91
Hochwollhagen	28.5	14	6.64	6.78	6.88	1.39	0.98	0.69
Hochwollhagen	11.5	13	5.81	6.12	5.94	1.53	0.73	1.18
Hochwollhagen	13.4	14	5.94	5.91	5.79	1.54	1.62	1.98
Hochwollhagen	11.0	12	5.77	5.54	5.52	1.54	2.23	2.28
Hochwollhagen	7.5	14	5.49	5.95	5.72	1.55	0.36	0.91
Hochwollhagen	11.1	12	5.77	5.66	5.59	1.56	1.88	2.10
Hochwollhagen	8.8	14	5.59	5.61	5.53	1.57	1.52	1.76
Hochwollhagen	12.6	15	5.86	5.89	5.76	1.61	1.53	1.90
Hochwollhagen	10.9	15	5.72	6.42	6.25	1.66	-0.01	0.34
Hochwollhagen	9.2	11	5.59	5.79	5.64	1.66	1.11	1.53
Hochwollhagen	11.8	13	5.78	5.96	5.81	1.68	1.19	1.61
Hochwollhagen	16.8	21	6.08	6.32	6.22	1.73	1.08	1.35
Hochwollhagen	22.5	14	6.31	6.56	6.56	1.84	1.13	1.14
Hochwollhagen	18.8	17	6.14	6.17	6.07	1.86	1.77	2.06
Hochwollhagen	18.3	13	6.11	6.30	6.21	1.87	1.34	1.59
Hochwollhagen	28.1	21	6.46	6.68	6.73	1.93	1.24	1.09
Hochwollhagen	35.9	16	6.59	6.66	6.64	1.97	1.71	1.76
Hochwollhagen	14.0	11	5.82	6.20	6.06	2.00	0.96	1.33
Hochwollhagen	11.1	14	5.62	5.55	5.53	2.00	2.22	2.28
Hochwollhagen	16.7	14	5.97	5.90	5.81	2.04	2.25	2.53
Hochwollhagen	8.2	11	5.38	5.36	5.41	2.06	2.12	1.96
Hochwollhagen	23.8	13	6.28	6.47	6.44	2.09	1.51	1.59
Hochwollhagen	6.4	10	5.21	5.41	5.40	2.12	1.51	1.55
Hochwollhagen	32.7	25	6.50	6.61	6.60	2.13	1.74	1.76
Hochwollhagen	16.7	15	5.92	5.94	5.84	2.19	2.13	2.42
Hochwollhagen	13.6	15	5.73	5.88	5.77	2.19	1.75	2.08
Hochwollhagen	15.1	14	5.81	6.12	5.99	2.24	1.34	1.70

Site	EC_a	Soil humidity	pH_{REF}	pH_{FE}	pH_{FEM2}	$Lime_{REF}$	$Lime_{FE}$	$Lime_{FEM2}$
	$mS\ m^{-1}$	wt.%	-	-	-		$Mg\ CaO\ ha^{-1}$	
Hochwollhagen	18.0	22	5.97	6.35	6.26	2.25	1.17	1.40
Hochwollhagen	21.1	14	6.09	6.31	6.24	2.35	1.67	1.89
Hochwollhagen	30.9	19	6.40	6.30	6.21	2.37	2.73	3.07
Hochwollhagen	19.6	14	6.01	6.29	6.21	2.38	1.54	1.78
Hochwollhagen	22.9	13	6.15	6.09	6.01	2.40	2.59	2.87
Hochwollhagen	24.5	17	6.19	6.40	6.35	2.46	1.79	1.93
Hochwollhagen	18.5	14	5.92	5.91	5.83	2.49	2.53	2.78
Hochwollhagen	13.3	14	5.61	5.60	5.58	2.51	2.54	2.60
Hochwollhagen	14.6	12	5.69	5.94	5.82	2.52	1.76	2.10
Hochwollhagen	7.4	12	5.13	5.87	5.66	2.66	0.53	1.05
Hochwollhagen	28.2	18	6.23	6.33	6.26	2.74	2.38	2.62
Hochwollhagen	12.3	14	5.45	5.37	5.47	2.82	3.09	2.75
Hochwollhagen	21.4	14	5.95	6.26	6.18	2.85	1.86	2.09
Hochwollhagen	16.4	15	5.69	5.60	5.61	2.87	3.18	3.15
Hochwollhagen	33.7	15	6.31	6.25	6.13	2.92	3.16	3.67
Hochwollhagen	22.0	15	5.93	5.78	5.75	3.02	3.56	3.68
Hochwollhagen	23.3	13	5.98	6.34	6.28	3.03	1.84	2.03
Hochwollhagen	16.3	14	5.62	5.64	5.63	3.09	3.03	3.05
Hochwollhagen	24.1	15	5.98	6.16	6.08	3.14	2.51	2.78
Hochwollhagen	11.9	11	5.32	5.86	5.73	3.17	1.47	1.85
Hochwollhagen	18.3	15	5.70	6.08	5.98	3.20	1.96	2.27
Hochwollhagen	16.4	14	5.59	5.78	5.72	3.22	2.58	2.77
Hochwollhagen	23.5	15	5.91	6.12	6.04	3.31	2.57	2.85
Hochwollhagen	21.0	16	5.79	6.00	5.92	3.35	2.63	2.89
Hochwollhagen	21.7	13	5.80	6.23	6.15	3.43	1.99	2.23
Hochwollhagen	17.2	14	5.56	5.73	5.69	3.49	2.89	3.02
Hochwollhagen	19.4	15	5.64	5.81	5.76	3.62	3.01	3.18
Hochwollhagen	17.9	14	5.54	5.97	5.88	3.70	2.24	2.52
Hochwollhagen	23.4	19	5.75	5.89	5.83	3.90	3.37	3.59
Hochwollhagen	22.7	20	5.72	5.57	5.61	3.90	4.50	4.32
Hochwollhagen	21.8	13	5.65	6.15	6.07	4.02	2.25	2.52
Hochwollhagen	16.6	13	5.38	6.01	5.90	4.03	1.90	2.22
Hochwollhagen	23.9	15	5.73	5.84	5.79	4.06	3.63	3.82
Hochwollhagen	21.4	11	5.59	6.01	5.93	4.18	2.65	2.92
Hochwollhagen	21.0	12	5.57	5.73	5.71	4.18	3.57	3.65
Suchsdorf	11.8	15	6.89	6.72	6.66	-0.64	-0.40	-0.30
Suchsdorf	12.5	15	6.72	6.79	6.77	-0.31	-0.42	-0.40
Suchsdorf	14.5	16	6.86	6.91	6.99	-0.31	-0.39	-0.51
Suchsdorf	12.1	14	6.64	6.53	6.41	-0.23	-0.04	0.20
Suchsdorf	13.1	15	6.71	6.84	6.86	-0.22	-0.43	-0.46
Suchsdorf	13.3	14	6.72	6.67	6.61	-0.22	-0.13	-0.03
Suchsdorf	15.3	16	6.85	6.69	6.67	-0.21	0.07	0.11

Site	EC_a	Soil humidity	pH_{REF}	pH_{FE}	pH_{FEM2}	$Lime_{REF}$	$Lime_{FE}$	$Lime_{FEM2}$
	mS m^{-1}	wt.%	-	-	-		Mg CaO ha^{-1}	
Suchsdorf	14.5	15	6.75	6.63	6.58	-0.13	0.09	0.20
Suchsdorf	16.4	15	6.85	6.71	6.72	-0.10	0.16	0.15
Suchsdorf	12.8	12	6.62	6.29	6.13	-0.10	0.56	0.93
Suchsdorf	14.9	17	6.73	6.72	6.71	-0.05	-0.03	-0.01
Suchsdorf	16.4	16	6.80	6.89	6.99	-0.02	-0.17	-0.33
Suchsdorf	14.9	15	6.71	6.75	6.75	-0.01	-0.08	-0.09
Suchsdorf	13.4	13	6.58	6.57	6.48	0.05	0.07	0.24
Suchsdorf	13.3	12	6.57	6.41	6.28	0.05	0.37	0.67
Suchsdorf	13.2	13	6.56	6.55	6.45	0.06	0.08	0.28
Suchsdorf	14.1	14	6.60	6.68	6.64	0.10	-0.05	0.02
Suchsdorf	13.9	16	6.58	6.54	6.45	0.11	0.19	0.38
Suchsdorf	13.3	14	6.51	6.28	6.13	0.17	0.66	1.02
Suchsdorf	16.6	15	6.71	6.69	6.69	0.18	0.22	0.22
Suchsdorf	13.7	15	6.50	6.42	6.30	0.24	0.41	0.69
Suchsdorf	13.6	14	6.49	6.33	6.19	0.25	0.59	0.92
Suchsdorf	15.3	14	6.59	6.55	6.48	0.27	0.35	0.50
Suchsdorf	16.4	17	6.65	6.38	6.28	0.28	0.88	1.12
Suchsdorf	15.8	14	6.61	6.50	6.42	0.29	0.52	0.70
Suchsdorf	16.5	14	6.62	6.75	6.77	0.35	0.09	0.04
Suchsdorf	15.2	12	6.54	6.34	6.22	0.36	0.81	1.09
Suchsdorf	18.2	15	6.70	6.48	6.42	0.37	0.87	1.01
Suchsdorf	15.0	16	6.51	6.37	6.25	0.40	0.71	0.98
Suchsdorf	16.5	16	6.59	6.85	6.93	0.41	-0.10	-0.23
Suchsdorf	18.9	16	6.71	6.54	6.51	0.41	0.80	0.88
Suchsdorf	16.4	16	6.57	6.79	6.83	0.44	0.00	-0.08
Suchsdorf	17.7	15	6.62	6.56	6.52	0.48	0.62	0.71
Suchsdorf	17.2	15	6.59	6.49	6.43	0.49	0.72	0.87
Suchsdorf	20.6	16	6.74	6.80	6.89	0.51	0.37	0.18
Suchsdorf	14.9	14	6.45	6.37	6.25	0.51	0.69	0.97
Suchsdorf	17.9	16	6.61	6.68	6.69	0.53	0.38	0.36
Suchsdorf	16.4	11	6.52	6.38	6.28	0.55	0.88	1.12
Suchsdorf	19.7	15	6.67	6.55	6.53	0.58	0.87	0.93
Suchsdorf	19.3	16	6.65	6.76	6.82	0.59	0.35	0.22
Suchsdorf	19.0	17	6.63	6.62	6.62	0.60	0.63	0.64
Suchsdorf	16.4	15	6.48	6.51	6.44	0.64	0.58	0.73
Suchsdorf	19.7	14	6.63	6.52	6.49	0.67	0.94	1.03
Suchsdorf	15.0	16	6.37	6.36	6.24	0.71	0.73	1.01
Suchsdorf	24.8	12	6.79	6.70	6.76	0.72	0.95	0.79
Suchsdorf	17.2	15	6.49	6.48	6.41	0.72	0.74	0.90
Suchsdorf	19.9	13	6.60	6.40	6.34	0.77	1.27	1.45
Suchsdorf	16.5	15	6.43	6.44	6.36	0.77	0.75	0.95
Suchsdorf	20.2	15	6.61	6.54	6.52	0.77	0.95	1.01

Site	EC_a	Soil humidity	pH_{REF}	pH_{FE}	pH_{FEM2}	$Lime_{REF}$	$Lime_{FE}$	$Lime_{FEM2}$
	mS m^{-1}	wt.%	-	-	-		Mg CaO ha^{-1}	
Suchsdorf	21.3	15	6.65	6.66	6.69	0.78	0.76	0.69
Suchsdorf	18.4	15	6.52	6.70	6.72	0.79	0.39	0.34
Suchsdorf	16.0	14	6.38	6.38	6.28	0.82	0.82	1.07
Suchsdorf	22.8	13	6.68	6.58	6.58	0.84	1.10	1.09
Suchsdorf	17.3	15	6.42	6.59	6.56	0.90	0.51	0.58
Suchsdorf	17.5	15	6.43	6.61	6.59	0.90	0.48	0.54
Suchsdorf	22.0	15	6.62	6.60	6.61	0.92	0.98	0.95
Suchsdorf	21.3	15	6.59	6.52	6.50	0.93	1.11	1.18
Suchsdorf	22.1	15	6.62	6.59	6.60	0.93	1.01	1.00
Suchsdorf	22.9	14	6.64	6.37	6.31	0.96	1.71	1.87
Suchsdorf	22.8	15	6.60	6.45	6.41	1.05	1.46	1.57
Suchsdorf	26.0	15	6.68	6.64	6.67	1.10	1.21	1.11
Suchsdorf	19.8	17	6.45	6.25	6.16	1.13	1.68	1.93
Suchsdorf	19.6	14	6.43	6.37	6.30	1.16	1.32	1.51
Suchsdorf	24.8	15	6.61	6.61	6.63	1.20	1.20	1.14
Suchsdorf	27.5	16	6.67	6.62	6.65	1.23	1.38	1.31
Suchsdorf	24.2	15	6.57	6.43	6.39	1.26	1.67	1.78
Suchsdorf	22.8	16	6.51	6.35	6.29	1.29	1.76	1.94
Suchsdorf	28.1	13	6.59	6.50	6.48	1.52	1.80	1.87
Suchsdorf	30.7	14	6.62	6.46	6.41	1.60	2.14	2.31
